# The Spaying of Mares

**Published:** 1897-06

**Authors:** W. L. Williams

**Affiliations:** Professor of Surgery and Obstetrics, New York State Veterinary College


// THE JOURNAL

OF

COMPARATIVE MEDICINE AND VETERINARY ARCHIVES.

VOL. XVIII. *JUNE*, 1897. No. 6.

### THE SPAYING OF MARES.

BY W. L. WILLIAMS, V.S.,
PROFESSOR OF SURGERY AND OBSTETRICS, NEW YORK STATE VETERINARY COLLEGE.

THE emasculation of domesticated animals, destroying their sexual power and instinct, has been freely practised for centuries, the reasons therefor varying with different species of animals, in different countries, and under diversified conditions. Most of these operations are so common that in many cases they are performed chiefly by stockowners, while others are left almost if not wholly to skilled veterinarians.

The spaying of mares has long been practised in Continental Europe, but there appears to be little modern literature upon the subject available to the American veterinarian. The earlier method of operating, which is now very rarely practised, consisted in making an incision in the flank of sufficient size to admit the operator's hand, through which the ovaries were removed. Later Charlier introduced the method of spaying the larger animals through the vagina, greatly decreasing the extent of the surgical wound, which, being protected by the vulvo-vaginal canal, rendered external influences less potent; and, added to this the more recent introduction of antiseptic and aseptic surgery, the spaying of mares has been included in the list of operations which may be undertaken by the veterinarian with every confidence in a successful issue.

In spite of the great advances in recent years in abdominal surgery, the spaying of mares has in this country been surrounded by an unwarranted fear, of such a degree that but few veterinarians attempt it, apparently believing the operation one of special difficulty and danger. As a consequence we have but few veterinarians that have spayed any considerable number of mares consecutively or in a manner which would serve as even a moderate test of the danger to the animal, or even give the surgeon the desired amount of experimental knowledge of the operation.

During my official connection as veterinarian with the Montana Agricultural Experiment Station a favorable opportunity occurred in the summer of 1896 for testing the effect of the operation on a larger scale than usual, and the experiment was consequently undertaken. Aside from the test as to the danger to the animal, and possible additional data as to the proper details of the operation, it was hoped to secure additional knowledge as to the economic value of the procedure as related to the rearing and handling of horses. Among the benefits hoped for were: First, increased docility of the animal; second, the prevention of the œstrum with the attendant nervousness, weakness, and repulsiveness; third, a safe mode of control in breeding.

As a general rule, it may be stated that emasculation of an animal increases its docility. This is more markedly true of the female than of the male. Sexual appetite being practically constant in the male, castration does not uniformly obliterate it, especially if the animal has not been emasculated until of adult age or near it; while in the female, with periodical sexual desire only, the obliteration of these periods of œstrum almost invariably destroys sexual instinct and renders the subject in the fullest sense neuter.

This end gained, we have secured the second class of benefits by doing away with the repulsive features of œstrum, avoiding at the same time the accompanying nervousness, irritability, and more or less incapacity for work.

The third incentive for the operation—that of the control or prevention of breeding—has a special application in Montana and other Rocky Mountain States, in that spaying is practically the only available method which can be employed to prevent unguarded mares from becoming pregnant not so much by valuable sires as by a low grade of mongrel horses, the progeny of which can but prove worthless. While Montana and perhaps most other Western States have laws against stallions running at large, they are quite inoperative, the ranges being constantly infested with mongrel stallions of the lowest type, so that mares are liable to impregnation at any time—this fact constituting one of the important causes of very marked degeneration of range-horses during the past few years. Moreover, there is a vast surplus of these semi-wild mongrel horses far beyond the demands of commerce, unsalable, and a serious nuisance to the ranges by consuming food which might otherwise be used by animals of distinct value. The operation would, then, if generally applied, soon reduce this class of horse to the legitimate number demanded by our commerce. Besides, it seems that in all probability the mare-gelding will prove more desirable than the gelding, the mares being generally of finer finish, are free from the repulsive and annoying accumulations of filth in the sheath of the penis of the gelding, and are cleaner in the stall, owing to the direction in which the urine is voided—the gelding unavoidably soiling his bedding, while the mare need not do so.

The instruments used in the operation consisted of a Reed injecting pump, vaginal dilator, like that used for spaying cows; a twenty-two inch Chassaignac écraseur with guarded ratchets, which could be worked within the vagina, and a concealed knife. The mares were in two lots, which we shall designate numerically. Lot No. 1 consisted of sixteen mares, of which one was a very weak, thin, half-starved mongrel three-year-old filly, procured but a day or two before operation, and fifteen grade French draught-mares, four to six years old, four of which were broken to saddle or harness, while eleven were range-mares, handled slightly as weanlings, since which time they had ranged unhandled and unguarded among the mountains. Except the three-year-old all were in prime condition, and presumably not pregnant. None of them were known to be vicious except one of the broken mares, which was at times balky, probably during the œstral periods. None of the animals were at the time receiving grain, and only the five broken mares had at any time been fed on grain, but had lived exclusively on the range, except as weanlings, they had had an allowance of hay.

The five broken mares were taken from the range on the evening of June 24, 1896, and placed in a bare corral with running water, where they could drink at will; and the eleven unbroken animals were placed in the same corral the following morning after a sharp drive of ten miles from their usual feeding-grounds. The five broken mares with five unbroken were operated upon on the evening of June 25th, and the remaining six on the morning of the 26th. The mares were led into a chute used for branding cattle, the unbroken ones having been caught with the lasso. They were secured by means of a halter to a post in front of the chute; bars were placed beneath the chest and abdomen and the tail drawn directly upward and made fast to a cross-beam, thus preventing the animal from lying down. The hind feet were secured by means of ropes to posts in front of and behind the animal, so that it could neither kick nor extend the feet forward in an attempt to lie down, while the possibility of rearing was prevented by tying a rope across the back. Two or three quarts of tepid water were then thrown into the rectum and the feces removed by an assistant; the vulva and perineum were washed and the vagina filled with a 1: 5000 solution of corrosive sublimate; the hands and arms washed in the same, and the instruments disinfected.

The vaginal tensor was then introduced with the hand, the prolongation fixed in the mouth of the uterus, the walls of the vagina rendered tense, the concealed knife carried into the vagina, opened, and an incision about one inch long made at the end of the vagina just above the mouth of the uterus, after which the knife was removed. The hand being again brought into the vagina, one or two fingers were pushed through the incision, followed by the other fingers of the hand in cone shape, the walls readily giving way and the entire hand pushed into the abdominal cavity. The écraseur was then introduced, guarded by the hand. By following the uterus, lying immediately beneath the incision, the cornua were soon reached, leading from it at almost right angles, soon ending, to be continued by the ligament of attachment, in which the ovary was readily and unmistakably found at a distance of two or four inches from the blunt end of the cornua. The chain of the écraseur was then placed over the ovary, and, holding the instrument and ovary securely with one hand, the instrument was tightened with the other, the ovary quickly crushed off, and carried out along with the écraseur. The crushing off of the ovary was practically the only step in which there was evidence of any considerable degree of pain, and this was not nearly so severe, judging by the struggles of the animal, as is seen in the castration of horses. The second ovary was removed in the same manner. Very little bleeding followed the operation. The animals were released without further procedure, and all confined in a meadow convenient for observation.

They were inspected twice daily on June 26th, 27th, 28th, and 29th, as closely as practicable with wild mares. There was some slight straining shown by most of the mares immediately after their release, and nearly all were noted lying down quietly. None of them at any time, so far as observed, showed any signs of severe pain, not nearly so severe as is commonly seen after the castration of stallions. The three-year-old mare, which as before stated, was in poor condition, in fact not fit to undergo an operation, seemed stiff and dull, but continued to graze daily. No discharge from the vagina was observed and no symptoms appeared to create alarm, and by the 29th she seemed somewhat improved. She was the first animal operated upon in the number, and my first operation on a mare, and in addition to her unfit condition, the emptying of her rectum was neglected, presuming that without grain the feces would be soft and pultaceous, but instead the rectum proved quite full of rather firm fecal matter, and in cutting upward toward it, and the vagina of the mare being *very much* thinner than that of the cow, the cut was made too deep and almost penetrated the rectum, a complication which probably did much to interfere with her recovery. Of the other mares none appeared to suffer noticeably, except one animal lay down much of the time, but when approached would jump to her feet, trot quickly away and commence to graze. Two or three others showed slight stiffness for twenty-four to forty-eight hours, but not sufficient to lead the ordinary observer to note anything amiss. When approached they all trotted briskly away, all grazed regularly, and apparently did not emaciate or lose weight.

In operating it was found that one mare was about seven months pregnant, and it was expected that she would abort, but she seemed to still be carrying her foal safely at the date of our last observation, and continued as well as the others, but later she lost her foal, whether by abortion or otherwise, is not known.

Lot No. 2 consisted of six farm-bred mares, aged from two to ten years, all broken, and at the time of operation were not being fed on grain; they were taken directly from the pasture for the operation, which was conducted the same as in Lot 1, on the 12th of August, 1896.

One of this lot, an aged mare with foal at her side, was apparently unwell for some weeks, while the others showed only slight effects of the operation for three or four days, and on September 8th, twenty-seven days after operating, the owner reported all completely recovered. The owner of Lot 1 reported all well on August 3d.

Of the twenty-two mares in the two lots, but twenty were successfully spayed, errors in two cases having necessitated the abandonment of the operation at an early stage. Both were fillies, one two, the other four years old. In these cases the incision through the vaginal walls was made too far posteriorly, behind the peritoneum, and instead of entering the abdomen led into the rectum. As the peritoneal sac remained intact, no serious harm came from the error, except that the operation was of necessity abandoned. The causes of the error were at least two, the vagina of the mare being very much thinner than that of the cow, to which the writer was accustomed; the incision was carried too far before danger was anticipated; but of greater importance was the essential difference in the relations between the vagina and rectum in the mare and cow. These have not, we believe, been distinctly pointed out, though probably other operators have noted the difference. The peritoneal covering on the superior surface of the vagina is not so extensive in the mare as in the cow, the rectum and vagina being in immediate contact until near the uterus, thus leaving but a very contracted field for operating, especially in fillies which have not bred. Moreover, it has been noted by others that manual exploration of the vagina of the mare causes it to dilate by becoming "erected," in which case it appears that the free part of the vagina assumes a position at right angles to the rectum. If, then, we follow the directions generally given, and make an incision in the vaginal walls two inches *posteriorly* to the os, we cut not into the peritoneal cavity, but into the rectum. Our error indicates clearly that the proper place for the incision is *above* and *immediately against* the os uteri, and that this is especially necessary in young fillies, in which the available field for operating is very contracted, yet ample, if these directions be followed.

Some writers direct that the vaginal incision should be complete, the entire thickness of the vaginal wall being cut through, and state that tearing through the peritoneal layer with the fingers should in no case be attempted, even though the knife need be introduced several times, declaring that tearing through this membrane is exceedingly liable to induce grave suppuration between the walls of the vagina and rectum. In a large proportion of the twenty cases above noted we cut only through the mucous and muscular coats, and completed the opening by tearing through the peritoneum with the fingers, yet, so far as we know, had no untoward results therefrom. It seems far more likely that the abscesses related by others were due rather to the same error we have recorded above, that is cutting either behind the peritoneum or just at its passage from the rectum to the vagina. Evidently such an error would tend to that result. We found it much easier to rupture the peritoneal coat of the mare with the fingers than it is in the cow.

The difficulty which some record, of a partial paralysis of the rectum after the operation, leading to retention of the feces and necessitating enemata, was not observed except in a minor degree with the two fillies in which our errors were made and the rectum wounded.

On the whole, our preparations were in some respects quite imperfect, especially the opportunities for careful asepsis. True, the animals were in the main very vigorous and in a climate not so prone to induce septic infection as many other regions; but, on the other hand, we feel that the disadvantages in our arrangements quite fully offset these.

We were forced to conclude from our experiment that the spaying of mares is neither difficult nor dangerous, and with some experience in castrating stallions under similar conditions we consider the spaying of mares the safer operation of the two. In each we open the abdominal cavity, in the stallion almost directly through the inguinal ring, in the mare quite indirectly through the long vagina.

With this experience before us, we have not hesitated in our free clinics at the New York State Veterinary College to spay mares as occasion requires. We recently had occasion to spay a vicious mare, dangerous to her driver on account of violent kicking, especially when in œstrum. Presented at the free clinic for operation, after a morning's work on a heavy ice wagon, without food, she was secured by placing her in an ordinary sling, her tail being secured upward to the sling's pulley, the hind feet secured forward and backward to rings placed in the walls, and the operation carried out under good aseptic precautions, without incident, except that the vulvo-vaginal walls contracted so forcibly about my arms as to render the operation difficult and cause my arms to ache painfully for seven or eight hours, although the operation was of but fifteen or twenty minutes' duration. The powerful contractions of the vulva constituted good evidence of an exalted perverted sexual excitability evidently closely related to the viciousness of the patient. The animal was immediately led home, a distance of one mile, fed moderately and exercised gently daily. The owner reported that her appetite was constantly good, she showed no stiffness or disinclination to move, or other signs of suffering from the effects of the operation, and after a period of ten days returned to her accustomed work, where she has since continued more docile and in better flesh than prior to emasculation.

It seems to us, therefore, that the spaying of mares is an operation quite warranted in general veterinary practice, less dangerous and painful than the castration of horses, and far less difficult than many of our more common operations. A more general introduction of the practice would greatly increase the value of many mares, especially those which are vicious, nervous, or repulsive during œstrum. Many of these are now bred in order to render them docile, a practice which is most unfortunate, as such mares are not at all suitable for breeding-purposes, tending strongly to reproduce their vicious characters in their progeny. In addition to the suggestions naturally emanating from our experience as here recorded, we may note that the method we have casually named, of securing mares for this operation by means of the ordinary horse-sling, is a most effectual and convenient one. With the tail secured upward to the sling-pulley, it is not only out of the way, but is of great value in preventing the animal from sitting down in the sling, while with the hind feet secure both backward and forward the mare can neither kick nor extend the members forward under the belly, but with tail and hind feet secure, and with the aid of the sling, the mare is readily kept in a posture favorable for operating.

